# Efficacy of amlodipine besylate and Valsartan for the treatment of mild to moderate hypertension

**DOI:** 10.1097/MD.0000000000016264

**Published:** 2019-06-28

**Authors:** Xiao-ou Wang, Wen Tian

**Affiliations:** Department of Geriatric Cardiology, The First Affiliated Hospital of China Medical University, Shenyang, China.

**Keywords:** amlodipine besylate, efficacy, hypertension, randomized controlled trial, safety, valsartan

## Abstract

**Background:**

Clinical researchers found that Amlodipine besylate and Valsartan (ABVS) can effectively treat mild to moderate hypertension (MMH). However, no study has systematically investigated its efficacy and safety for patients with MMH. Thus, present study will systematically assess the efficacy and safety of ABVS for patients with MMH.

**Methods:**

MEDICINE, Cochrane Library, EMBASE, Ovid, PsycINFO, Web of Science, Allied and Complementary Medicine Database, and China National Knowledge Infrastructure will be searched for literatures related to the topic from inception to the present without language limitations. All randomized controlled trials that assess the efficacy and safety of ABVS for patients with MMH will be considered for inclusion. Two researchers will independently select study, extract data, and assess risk of bias for all eligible studies.

**Results:**

The primary outcome includes the change of seated diastolic blood pressure. The secondary outcomes consist of the change of seated systolic blood pressure, health-related quality of life, and the tolerability.

**Conclusions:**

The results of this study will summarize the latest evidence on ABVS for the treatment of MMH.

**Ethics and dissemination:**

This study does not need ethical approval, because it will not use individual data. The results of this study are expected to be published at peer-reviewed journals.

**PROSPERO registration number:**

PROSPERO CRD42019133123.

## 1 Introduction

Hypertension is a major risk factor for patient with cardiovascular disease and stroke,^[1–3]^ which can increase the morbidity and mortality for those patients.^[4–6]^ It has been reported that hypertension was 69% in patients who experience first heart attack, and 77% in patients who were diagnosed with fist strokes.^[6–8]^ Previous study also reported that the effective reduction of blood pressure is closely related to the decrease of cardiovascular disease and stroke.^[8]^ Additionally, the antihypertensive therapy is directly associated with the reduction of blood pressure.^[9,10]^

It has been estimated that most patients with hypertension are not adequately controlled by using antihypertensive monotherapy.^[11–14]^ Thus, combination therapy is recommended to treat this condition, especially for the combination of an angiotensin II receptor blocker and a calcium channel blocker.^[15,16]^ However, the different combined therapies have significantly different efficacy and safety.^[17,18]^

The efficacy and safety of the combination of amlodipine besylate and valsartan (ABVS) has been well established for mild to moderate hypertension (MMH).^[19–23]^ However, no study has systematically assessed the efficacy and safety of ABVS for patients with MMH. Therefore, in this study, we will systematically investigate the efficacy and safety of ABVS for patients with MMH.

## 2 Methods and analysis

### 2.1 Study registration

This study has been registered on PROSPERO (CRD42019133123), and we have reported it based on the guidelines of Preferred Reporting Items for Systematic Reviews and Meta-Analysis (PRISMA) Protocol statement.^[24]^

### 2.2 Eligibility criteria for study selection

#### 2.2.1 Types of studies

We will only consider randomized controlled trials (RCTs) of ABVS for patients with MMH. However, the studies of non-RCTs and quasi-RCTs will be excluded.

#### 2.2.2 Types of interventions

The experimental intervention includes ABVS. However, the treatment of amlodipine besylate or valsartan alone, or combined with other treatments will all be excluded.

The control treatment can be any kinds of therapies except ABVS.

#### 2.2.3 Types of participants

Patients with MMH will be included without any limitations of race, sex, and age.

#### 2.2.4 Types of outcome measurements

The primary outcome is the change of seated diastolic blood pressure. The secondary outcomes comprise of the change of seated systolic blood pressure; health-related quality of life, as measured by EuroQol scale or the 36-Item Short Form Health Survey, or any other relevant scales; as well as any expected or unexpected tolerability.

### 2.3 Search strategy

#### 2.3.1 Electronic databases search

We will identify the following electronic databases for relevant studies from inceptions to the April 20, 2019: MEDICINE, Cochrane Library, EMBASE, Ovid, PsycINFO, Web of Science, Allied and Complementary Medicine Database, and China National Knowledge Infrastructure without language restrictions. We will only include RCTs that evaluate the efficacy and safety of ABVS for patients with MMH. The detailed search strategy for MEDLINE is presented in Table 1. We will also apply similar search strategy to the other electronic databases.

**Table 1:**
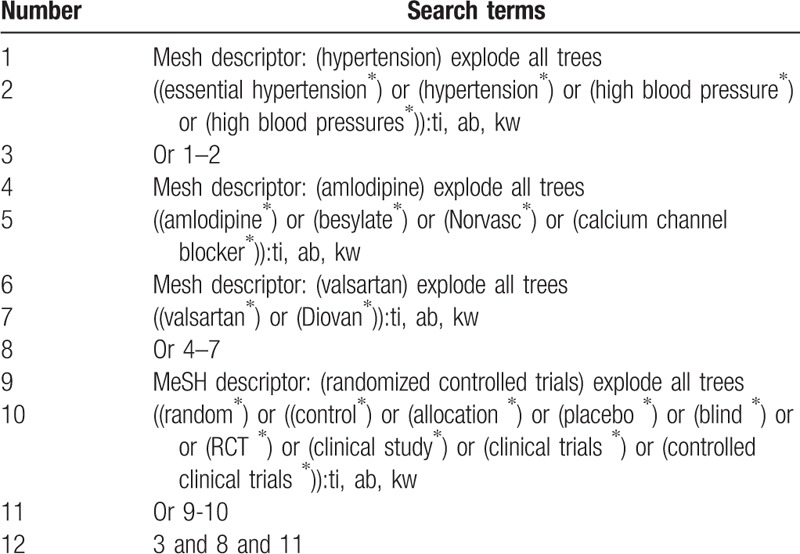
Search strategy sample of Cochrane Library.

#### 2.3.2 Other literature sources search

Other literature sources including clinical registry, reference lists of included RCTs, as well as conference proceedings will also be considered to search.

### 2.4 Literature selection

Two reviewers will independently select all the literatures by scanning titles and abstracts initially, and then exclude the irrelevant studies. After that, all potential eligible studies will be read by full text for further assessment. Any disagreements regarding the study selection between 2 reviewers will be solved by a third reviewer through discussion. The results of study selection will be shown in PRISMA flowchart.

### 2.5 Data extraction

All data will be extracted according to the pre-designed data extraction sheet. Two reviewers will independently extract all the information and data. A third reviewer will be participated to solve any disagreements regarding data extraction between the 2 reviewers.

The extracted information comprises of general information (such as title, first author, year of publication, eligibility criteria, etc); study methods (such as sample size, randomization, concealment, blinding, etc); treatment schedules (such as medication names, dosage, duration, etc); and outcomes (such as primary, secondary, and safety outcomes).

### 2.6 Dealing with missing data

Any missing data or insufficient information will be required from the primary authors by email. We will analyze the current available data if that insufficient information is not achievable.

### 2.7 Methodological quality assessment

Methodological quality for each study will be assessed by using Cochrane risk of bias tool. It consists of 7 domains, and each 1 will be categorized as high, unclear or low risk of bias. Two reviewers will independently assess the methodological quality for each trial. Any divergences between 2 reviewers will be settled down through discussion with a third reviewer.

### 2.8 Statistical analysis

RevMan 5.3 software will be utilized to analyze the data and to conduct statistical analysis. We will express continuous data as mean difference and 95% confidence intervals, and dichotomous data as risk ratio and 95% confidence intervals. *I*^2^ test will be used to check heterogeneity among all eligible studies. *I*^2^ ≤ 50% indicates a minor heterogeneity. Then, a fixed-effect model will be utilized to pool the data, and meta-analysis will be performed. *I*^2^ > 50% indicates a significant heterogeneity, and a random-effect model will be used to pool the data. We will also carry out subgroup analysis base on the different characteristics, treatment schedules, and outcome measurements. If significant heterogeneity still can be identified after subgroup analysis, data will not be pooled, and only a narrative summary will be reported.

In addition, sensitivity analysis will also be carried out to identify the robustness and stability of outcome results by removing low quality eligible studies. If more than 10 eligible RCTs are included, we will also conduct funnel plot and Egger regression test to check potential reporting bias.^[25,26]^

## 3 Discussion

As we know, monotherapy is often used for antihypertensive treatment in patients with MMH.^[27]^ However, most patients can not achieve promising efficacy for blood pressure control.^[27]^ Thus, the use of combination therapy is utilized to treat such condition.

Although several previous studies have explore the efficacy of the combination of ABVS for the treatment in patients with MMH,^[19–23,28]^ no study has been systematically addressed for assessing the efficacy and safety of ABVS for patients with MMH. Thus, this study will systematically explore the efficacy and safety of ABVS for MMH. The results of this study are expected to summarize the latest evidence regarding the efficacy and safety of ABVS for MMH. Its findings may also provide helpful evidence for clinician.

## Author contributions

**Conceptualization:** Xiao-Ou Wang, Wen Tian.

**Data curation:** Xiao-Ou Wang, Wen Tian.

**Formal analysis:** Xiao-Ou Wang, Wen Tian.

**Investigation:** Wen Tian.

**Methodology:** Xiao-Ou Wang.

**Project administration:** Wen Tian.

**Resources:** Xiao-Ou Wang.

**Software:** Xiao-Ou Wang.

**Supervision:** Wen Tian.

**Validation:** Xiao-Ou Wang, Wen Tian.

**Visualization:** Xiao-Ou Wang, Wen Tian.

**Writing – original draft:** Xiao-Ou Wang, Wen Tian.

**Writing – review & editing:** Xiao-Ou Wang, Wen Tian.
